# Antigenic drift and immunity gap explain reduction in protective responses against influenza A(H1N1)pdm09 and A(H3N2) viruses during the COVID-19 pandemic: a cross-sectional study of human sera collected in 2019, 2021, 2022, and 2023

**DOI:** 10.1186/s12985-024-02326-w

**Published:** 2024-03-06

**Authors:** Even Fossum, Andreas Rohringer, Torstein Aune, Kjersti Margrethe Rydland, Karoline Bragstad, Olav Hungnes

**Affiliations:** 1 Division of Infection Control, Department of Virology, Norwegian Institute of Public Health, PO Box 222 Skøyen, 0213 Oslo, Norway; 2 Division of Infection Control, Department of Vaccines, Norwegian Institute of Public Health, PO Box 222 Skøyen, 0213 Oslo, Norway

**Keywords:** Influenza, Serology, Antibody, A(H1N1)pdm09, A(H3N2), Antigenic drift, Immunity gap

## Abstract

**Background:**

Non-pharmaceutical interventions implemented during the COVID-19 pandemic resulted in a marked reduction in influenza infections globally. The absence of influenza has raised concerns of waning immunity, and potentially more severe influenza seasons after the pandemic.

**Methods:**

To evaluate immunity towards influenza post-COVID-19 pandemic we have assessed influenza A epidemics in Norway from October 2016 to June 2023 and measured antibodies against circulating strains of influenza A(H1N1)pdm09 and A(H3N2) in different age groups by hemagglutination inhibition (HAI) assays in a total of 3364 serum samples collected in 2019, 2021, 2022 and 2023.

**Results:**

Influenza epidemics in Norway from October 2016 until June 2023 were predominately influenza As, with a mixture of A(H1N1)pdm09 and A(H3N2) subtype predominance. We did not observe higher numbers of infections during the influenza epidemics following the COVID-19 pandemic than in pre-COVID-19 seasons. Frequencies of protective HAI titers against A(H1N1)pdm09 and A(H3N2) viruses were reduced in sera collected in 2021 and 2022, compared to sera collected in 2019. The reduction could, however, largely be explained by antigenic drift of new virus strains, as protective HAI titers remained stable against the same strain from one season to the next. However, we observed the development of an immunity gap in the youngest children during the pandemic which resulted in a prominent reduction in HAI titers against A(H1N1)pdm09 in 2021 and 2022. The immunity gap was partially closed in sera collected in 2023 following the A(H1N1)pdm09-dominated influenza seasons of 2022/2023. During the 2022/2023 epidemic, drift variants of A(H1N1)pdm09 belonging to the 5a.2a.1 clade emerged, and pre-season HAI titers were significantly lower against this clade compared to the ancestral 5a.2 clade.

**Conclusion:**

The observed reduction in protective antibodies against A(H1N1)pdm09 and A(H3N2) viruses post COVID-19 is best explained by antigenic drift of emerging viruses, and not waning of antibody responses in the general population. However, the absence of influenza during the pandemic resulted in an immunity gap in the youngest children. While this immunity gap was partially closed following the 2022/2023 influenza season, children with elevated risk of severe infection should be prioritized for vaccination**.**

**Supplementary Information:**

The online version contains supplementary material available at 10.1186/s12985-024-02326-w.

## Background

Non-pharmaceutical interventions (NPI) introduced in 2020 to limit the spread of SARS-CoV-2 drastically reduced the global spread of respiratory viruses and terminated the 2019/2020 influenza season early in Norway [1]. In Norway, NPIs consisted of temporary closing of schools, remote education, recommendation to work from home, social distancing and closing/restricting access to restaurants/bars and indoor sport and cultural events [2]. There were hardly any confirmed cases of influenza virus infections from April/May 2020 until December 2021, after which there was a resurgence in influenza infections that peaked around week 14 2022. The prolonged absence of influenza has raised concerns of whether waning immunity would result in more extensive influenza epidemics as respiratory viruses have returned following the removal of NPIs. Indeed, several countries, including Norway, have observed out-of-season resurgence of respiratory viruses such as Respiratory Syncytial virus with the relaxation of NPIs [3–6], while influenza infections returned in many countries towards the end of 2021 [7, 8]. However, it is not clear how the resurgence in respiratory viruses correlate with potential changes in population immunity over the pandemic.

The level of protective immunity against influenza viruses in the population is a result of vaccinations and previous infections (reviewed in [9]). However, the nature of the immune response induced by infection or vaccination can vary extensively. Natural influenza infection can induce long lasting immune response consisting of humoral and cellular immunity against several viral antigens. For instance, during the influenza A(H1N1)pdm09 pandemic in 2009 it was observed that older individuals were better protected against infection, most likely due to previous exposure to antigenically similar A(H1N1) strains more than 50 years prior [10, 11]. Indeed, HAI assay performed on sera collected prior to the 2009 pandemic observed the highest seropositivity towards A(H1N1)pdm09 virus in the > 80 years age group [12], and B cells specific for the 1918 A(H1N1) virus have been isolated in elderly donors nearly 90 years after the pandemic [13]. In contrast, vaccination is thought to induce a more short-lived and strain-specific antibody response, predominantly directed against the hemagglutinin (HA) surface antigen [9]. As a consequence, there are reports of intra-seasonal waning of vaccine induced protection [14, 15], and laboratory studies indicating that antibody levels are significantly reduced within 6 months [16]. However, antibody levels are still elevated after 6 months compared to pre-vaccination samples [17], and vaccination remains the best option for reducing the number of deaths and hospitalizations from seasonal influenza.

While immune responses may provide protection from infection, they also exerts evolutionary pressure on the virus to mutate in order to evade existing immunity – a process referred to as antigenic drift. Consequently, the emergence of strains with a new antigenic profile may render a larger part of the population susceptible to infection and disease. To evaluate immunity against influenza in Norway, the National Influenza Centre (NIC Norway) at the Norwegian Institute of Public Health (NIPH) conducts an annual collection of residual sera from laboratories across the country from a geographical and age-representative selection of the population [18, 19]. The serum samples are collected in August each year and analyzed by HAI assays for protective antibodies against influenza viruses that have circulated in previous seasons or are expected to circulate in the coming season. HAI assays have long been established as a correlate of protection in humans [20, 21], and it is generally established that an HAI titer of 1:40 or more will reduce the risk of influenza A infection by 50% (reviewed in [22]). Here we present a summary of influenza A infections from October 2016 to May 2023, and HAI titers against A(H1N1)pdm09 and A(H3N2) strains in sera collected in 2019, 2021, 2022, and 2023.

## Methods

### Collection of serum samples

Anonymized residual sera were collected in August/September in 2019, 2021, 2022 and 2023 from medical laboratories across Norway as previously described [12, 23]. In total, 3364 sera were selected for analysis by HAI. Sera were collected to be representative of the Norwegian population in terms of geography and age distribution. The mean age of the collected sera were 34 years and 56.1% were from females. Characteristics of the collected sera for each year are presented in Table 1. Due to an increased workload related to the COVID-19 pandemic, serum samples from 2020 were not analyzed for antibodies against influenza.

**Table 1:** Characteristics of collected serum samples

	2019	2021	2022	2023	Total
Number of sera tested	1054	657	1197	456	3364
Sex- female (%)	54,7	53,4	56,8	60,0	56,1
Avg. age (Years)	33	34,9	34,4	34	34
Age group:					
0–4 years (N)	113	48	90	40	291
5–14 years (N)	187	107	210	79	583
15–24 years (N)	171	114	204	77	566
25–59 years (N)	375	250	455	169	1249
60 + years (N)	208	138	238	91	675

### Quantitating influenza infections and vaccine coverage

Influenza detections from all microbiology laboratories in Norway were until 2020 reported directly to the NIC on a weekly basis, and after this in real-time via the Norwegian microbiology laboratory database (MSIS laboratory database). The aggregated number of influenza infections from October 2016 until May 2023 were obtained by combining data from the two registries. Since 2009, more laboratories test for A(H1N1)pdm09 than for A(H3N2), hence a larger portion of the patient samples will get a positive subtyping result for H1 than for H3. Used raw, the national subtyping data will underestimate the number of A(H3N2) infections and overestimate A(H1N1)pdm09. To correct for this bias, the proportion of subtype A(H1N1)pdm09 to A(H3N2) infections were calculated based only on samples that were tested against both subtypes, and the proportions projected onto the total number of laboratory confirmed influenza A infections.

To determine the number of confirmed influenza A infections per 100.000 by age group, the population number within each age group were obtained from Statistics Norway for Q1 2022 and Q1 2023 [24]. Influenza A infections data were obtained from the MSIS database on 4. October 2023 for the period week 1 – 26 2022 (predominantly A(H3N2)) and week 40 2022 – week 22 2023 (predominantly A(H1N1)pdm09).

Vaccine coverage for the different age groups used in the manuscript were obtained from the Norwegian Immunization Registry SYSVAK.

### A(H1N1)pdm09 and A(H3N2) PCR

Various PCR methods were used for influenza A and A(H1N1)pdm09 virus testing in the primary testing laboratories, while the majority of A(H3N2) subtyping were performed at the NIC. In the NIC, identification of A(H1N1)pdm09 was done using a protocol developed by the Robert Koch Institut, Germany [25], whereas A(H3N2) subtyping was done using a PCR developed by the Influenza Division, Centers for Disease Control and Prevention, Atlanta, GA, USA (https://www.cdc.gov/ncird/flu.html). In addition, influenza A and A(H1N1)pdm09 virus testing were verified in the NIC by PCR and a subset of samples selected for whole-genome sequencing of full-length viral RNA segment amplicons by Oxford Nanopore technology (nanopore baroding with PCR amplifications adopted from Zhou. et al. [26]).

### Generating phylogenetic trees of A(H1N1)pdm09 and A(H3N2) viruses

Phylogenetic trees were generated using the software Molecular Evolutionary Genetics Analysis (MEGA) version 11 [27]. For the A(H1N1)pdm09 and A(H3N2) HA gene phylogenetic trees in Additional file 1: Fig. S2, evolutionary history was inferred using the Neighbor-Joining method [28], with evolutionary distance calculated the Maximum Composite Likelihood method[29]. For the A(H1N1)pdm09 tree A/Christchurch/16/2010 was applied as root, while A/Texas/50/2012 was applied as root in the A(H3N2) tree. All the HA gene sequences of A(H1N1)pdm09 and A(H3N2) strains in Additional file 1: Fig. S2 were obtained from the GISAID EpiFlu database [30] with the accession numbers; EPI_ISL_79721 (A/Christchurch/16/2010), EPI_ISL_199532 (A/Michigan/45/2015), EPI_ISL_306335 (A/Brisbane/02/2018), EPI_ISL_332059 (A/Darwin/6/2018), EPI_ISL_377080 (A/Guangdong-Maonan/SWL1536/2019), EPI_ISL_401903 (A/Victoria/2570/2019), EPI_ISL_6424984 (A/Sydney/5/2021), EPI_ISL_122006 (A/Texas/50/2012), EPI_ISL_292575 (A/Kansas/14/2017), EPI_ISL_285898 (A/Singapore/INFIMH-16–0019/2016), EPI_ISL_710475 (A/Cambodia/e0826360/2020), EPI_ISL EPI_ISL_2233240 (A/Darwin/9/2021).

The phylogenetic analysis of Norwegian A(H1N1)pdm09 viruses sequenced during the 2022/2023 season was performed in BioNumerics version 8.0 by generating a Maximum parsimony tree of HA sequences, including reference strains and vaccine strains for the southern and northern hemisphere.

### Propagation of influenza virus

With the exception of A/Norway/25089/2022 (H1N1)pdm09 which was propagated in MDCK cells, the influenza viruses for the HAI assay were egg isolates kindly provided by the Worldwide Influenza Centre at the Francis Crick Institute, UK, and propagated in the allantoic cavity of embryonated hen’s eggs following the WHO GISRS Manual for the laboratory diagnosis and virological surveillance of influenza [31].

### Hemagglutination inhibition assay (HAI)

Serum antibody titers were determined using an HAI assay, as previously described [32]. In short, aliquots of the collected sera were treated with receptor destroying enzyme (RDE) over night (ON) at 37°C, followed by inactivation by incubating for 30 min at 56°C. Next, the sera were serially diluted two-fold in 96-well plates starting at dilution 1:10 in PBS pH 7.2, resulting in a final volume of 25 μl pr well. Viral antigen in the form of different strains of influenza virus was subsequently added to the wells at a concentration 6–8 hemagglutinating units (HAU) pr 25 μl. Diluted sera and influenza virus were incubated for 1h at room temperature, before addition of turkey red blood cells (RBC) as indicator cells at a concentration of 0.25% in PBS with BSA. The HAI titer was determined as the serum dilution factor that produced complete inhibition in the assay. An HAI titer of 40 or higher against a particular influenza virus strain is associated with reduced risk for infection [20, 21]. For calculations of geometric mean titers, sera with titers < 10 were assigned an HAI titer of 5. Sera collected in 2019 were tested against the A(H1N1)pdm09 strains A/Michigan/45/2015 and A/Brisbane/2/2018, while sera collected in 2021, 2022 and 2023 were tested against A/Victoria/2570/2019. In addition, sera from 2019 were tested against A(H3N2) strains A/Singapore/19/2016 and A/Kansas/14/2017, sera from 2021 were tested against A/Cambodia/e0826360/2020 and A/Darwin/9/2021 and sera from 2022 and 2023 were tested against A/Darwin/9/2021.

### Statistics

HAI titers in Fig. 3 and Fig. 4 were plotted as reverse cumulative plots [33], while HAI titers in Fig. 5 were plotted as geometric mean with 95% confidence interval. Significant difference in HAI titers between two groups were calculated using a Mann–Whitney test or a Wilcoxon matched-pairs signed rank test using GraphPad Prism 9.

## Results

**Fig. 1:**
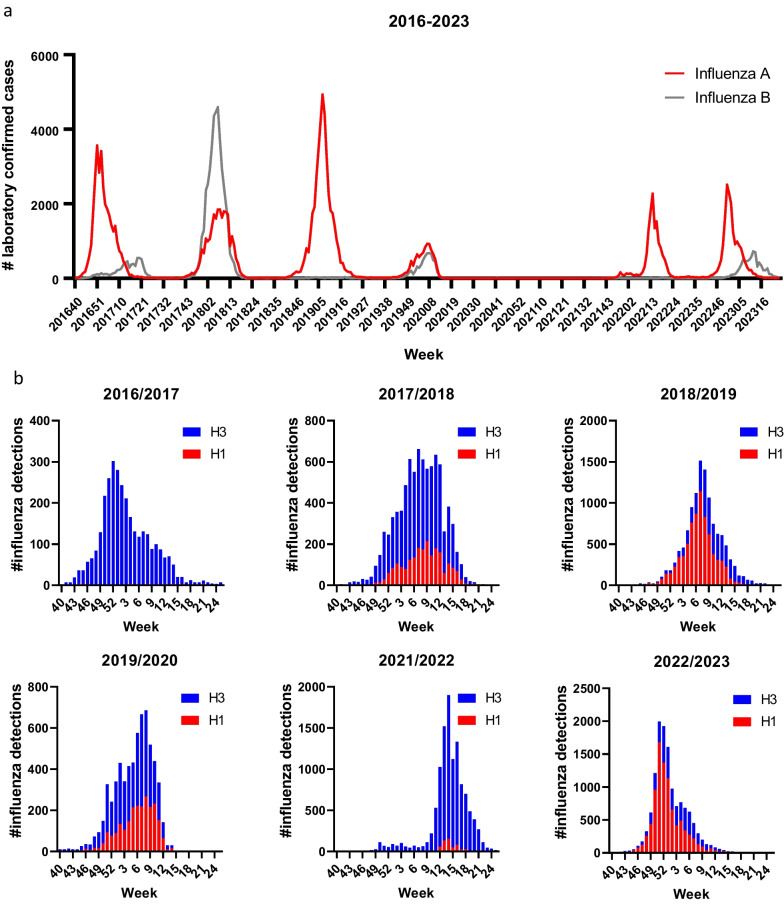
Influenza epidemics in Norway from October 2016 to May 2023. **A** The number of laboratory confirmed influenza A and B infections were extracted from the Norwegian influenza surveillance data generated by NIPH and the regional laboratories. **B** The proportions of A(H1N1)pdm09 and A(H3N2) among influenza A infections were estimated from patient samples tested against both subtypes, and these weekly frequencies extrapolated to the total number of detected influenza A infections. Each influenza season is indicated with a start in week 40 and end in week 25 the following year

To assess the influenza epidemics in Norway before and after the COVID-19 pandemic, the number of confirmed influenza A and B cases were plotted from October 2016 until June 2023 (Fig. 1A). While the 2016/2017 and 2018/2019 seasons were dominated by influenza A, the 2017/2018 season had an overweight of influenza B cases. The 2019/2020 influenza season was mild and had an even mix of influenza A and B detections. The 2019/2020 season progressed largely as normal, with a start in October/November 2019 and a peak February 2020, although the number of infections quickly declined in March 2020 with the introduction of contact-reducing measures to limit the spread of SARS-CoV-2 (Fig. 1A)[1]. During the 2020/2021 season NIPH only registered 11 confirmed influenza A infections and 11 influenza B cases with many of them being imported, and influenza viruses were largely absent from April 2020 until December 2021. After low levels of influenza infections during winter 2021/2022 there was a late influenza epidemic that started in March 2022, after all non-pharmaceutical interventions against COVID-19 had ended in February 2022. The influenza A epidemic of the 2022/2023 season started relatively early and peaked around Christmas 2022, followed by a smaller increase in influenza B infections in February to April 2023. Despite the absence of influenza viruses for almost two years, the two influenza epidemics that have occurred after start of the COVID-19 pandemic have not resulted in a higher number of confirmed infections compared to the influenza seasons before the pandemic.

Influenza A subtype distribution indicates that the 2016/2017 and 2021/2022 seasons were dominated by the influenza A(H3N2) subtype, while the 2018/2019 and 2022/2023 seasons had a higher number of A(H1N1)pdm09 subtype infections (Fig. 1B). The 2019/2020 season was more mixed between A(H1N1)pdm09 and A(H3N2) infections, while the 2017/2018 season consisted predominantly of B/Yamagata infections in addition to A(H3N2) infections (Fig. 1A-B, Additional file 1: Figure S1). Indeed, since the A(H1N1)pdm09 pandemic in 2009/2010, influenza A viruses have predominated in most seasons, with only the 2010/2011 and 2017/2018 seasons being influenza B dominated (Additional file 1: Figure S1).

### Protective immunity against influenza A viruses

**Fig. 2:**
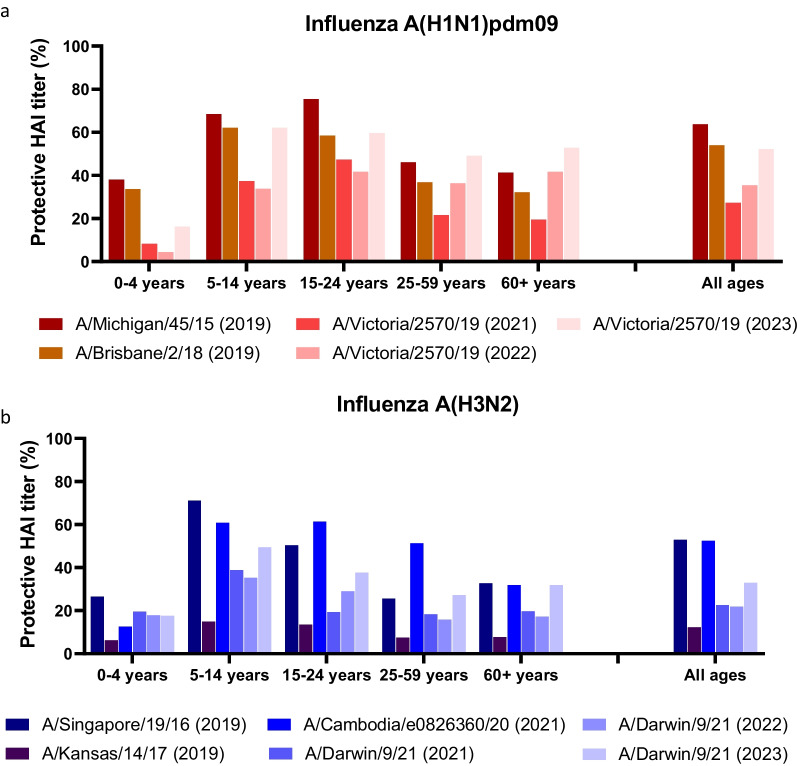
Protective antibodies against influenza A in sera from 2019, 2021, 2022 and 2023. Residual sera collected in August 2019 (*n* = 1054), 2021 (*n* = 657), 2022 (*n* = 1197) and 2023 (*n* = 456) were evaluated in HAI assay against **A** H1N1pdm09 strains A/Michigan/45/2015, A/Brisbane/2/2018 or A/Victoria/2570/2019 or **B** H3N2 strains A/Singapore/19/2016, A/Kansas/14/2017, A/Cambodia/e0826360/2020 or A/Darwin/9/2021. Sera were considered protective if HAI titers were ≥ 40, and the percentage of protective-titre sera plotted in different age groups

To assess the level of immunity towards influenza before and after the COVID-19 pandemic, we compared HAI titers in serum samples collected in 2019, 2021, 2022 and 2023. Serum samples were collected to be representative of the Norwegian population in terms of geography and age distribution (Table 1) and were analyzed by HAI assays against influenza strains that were circulating, or were expected to circulate the following influenza season, and that were part of previous or current seasonal influenza vaccines (Fig. 2A-B). Given the dominance of influenza A from fall 2016 until spring 2023 we focused on immunity towards A(H1N1)pdm09 and A(H3N2)(Fig. 2).

Sera was considered protective if HAI titers were ≥ 40, and the percentage of protective sera plotted for each influenza strain (Fig. 2A). For sera collected in 2019, 63.7% of all serum samples had protective HAI titers against A/Michigan/45/2015 (H1N1pdm09, Clade 6B.1). There was a reduction to 53.9% protective titers against A/Brisbane/2/2018 of the Clade 6B.1A1 in the same sera (Additional file 1: Figure S2A)[34]. There was a more pronounced reduction in protective HAI titers to 27.3% in sera from 2021, which were tested against A/Victoria/2570/2019. However, this reduction may also relate to further antigenic drift of the A/Victoria/2570/2019 strain which belongs to the 6B.1A.5a.2 clade (Additional file 1: Figure S2A)[35]. In sera collected in 2022 there was an increase in the percentage of protective HAI titers to 35.4% against A/Victoria/2570/2019, even though there was very little circulation of A(H1N1)pdm09 virus during the 2021/2022 season. A/Victoria/2570/2019 was, however, included in the seasonal influenza vaccine that was administered in the fall/winter of 2021 and in the fall/winter of 2022 (Fig. 2A)[36]. Following the A(H1N1)pdm09 dominated 2022/2023 influenza season we observed a further increase in the percentage of protective HAI-titers against A/Victoria/2570/2019 to 52.2% in sera collected in 2023.

For influenza A(H3N2), 52.9% of the sera collected in 2019 had protective HAI titers against A/Singapore/19/2016, while only 12.3% had protective HAI titers against A/Kansas/14/2017 (Fig. 2B). These two strains belong to different clades, 3C.2a1 and 3C.3 respectively, which explains the large difference in protective HAI titers (Additional file 1: Figure S2B). In sera collected in 2021, 52.4% had protective titers against A/Cambodia/e0826360/2020 which belongs to a continuation of the 3C.2a1 clade, this indicates that protective HAI titers against this clade have remained quite stable over the pandemic. In contrast, only 22.6% of serum samples from 2021 had protective HAI titers against A/Darwin/9/2021 of the 3C.2a1b.2a.2 clade (Additional file 1: Figure S2B). Protective titers against A/Darwin/9/2021 remained stable at 21.9% of sera collected in 2022, while increasing to 32.9% of collected sera in 2023 (Fig. 2B). In contrast to the A/Victoria/2570/2019(H1N1) strain, the A/Darwin/9/2021(H3N2) strain was first included in the northern hemisphere seasonal vaccine for 2022/2023, which may have contributed to the increase in protective responses from 2022 to 2023.

### HAI responses in different age groups

When breaking the HAI data into age groups we observed that in the 25–59 years and 60 + years groups, the percentage of sera with protective HAI tiers against A(H1N1)pdm09 was reduced in sera from 2021, compared to sera from 2019. Similarly to the percentages observed for all serum samples, there was an increase in sera with protective titers from 2021 to 2022, which was further increased in 2023. (Fig. 2). In the 0–4 years age group there was a marked reduction in the percentage of protective HAI titers against A(H1N1)pdm09 from 33.6% against A/Brisbane/2/2018 in 2019 to 8.3% against A/Victoria/2570/2019 in 2021. There was a further reduction to 4.4% against A/Victoria/2570/2019 in 2022, likely reflecting the fact that most children born after the 2018/2019 influenza season were immunologically naïve to A(H1N1)pdm09 viruses. Following the A(H1N1)pdm09 dominated influenza season of winter 2022/2023, there was an increase in protective titers to 16.2% against A/Victoria/2570/2019. For the 5–14 years and 15–24 years age groups the percentage of protective sera was comparable or higher than those observed for the combined age groups.

**Fig. 3:**
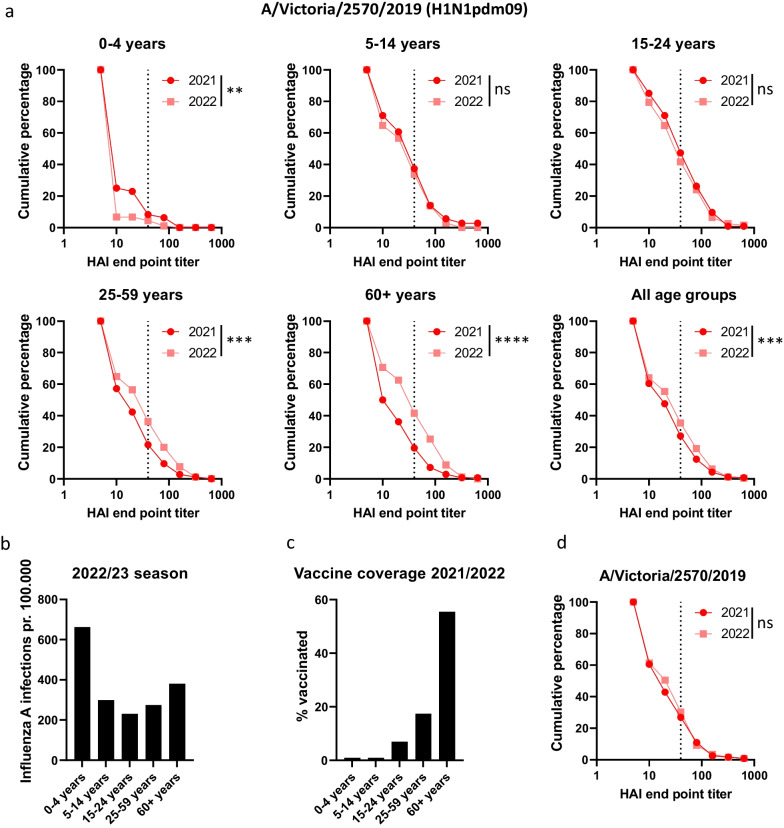
HAI titers against H1N1pdm09 A/Victoria/2570/2019 in sera from 2021 and 2022 in different age groups. **A** Reverse cumulative plots were generated from the HAI titers against A/Victoria/2570/19 from 2021 and 2022 for the age groups 0–4 years, 5–14 years, 15–24 years, 25–59 years and 60 + years. The dotted line indicates 50% protective HAI titer of 40. **B** Number of detected influenza A infections per 100.000 individuals in the different age groups for the period week 40 2022 to week 22 2023. **C** Vaccine coverage in the general population obtained from the Norwegian Immunization Registry SYSVAK for the different age groups. **D** Reverse cumulative plots of HAI titers in a panel of 119 sera collected in 2021 and first tested against A/Victoria/2570/2019 in 2021 and repeat tested in 2022 to verify reproducibility. **A** Significant differences were calculated by a two-tailed Mann–Whitney test. **p < 0.01, ***p < 0.001 and****p < 0.0001, ns = no significant difference

**Fig. 4:**
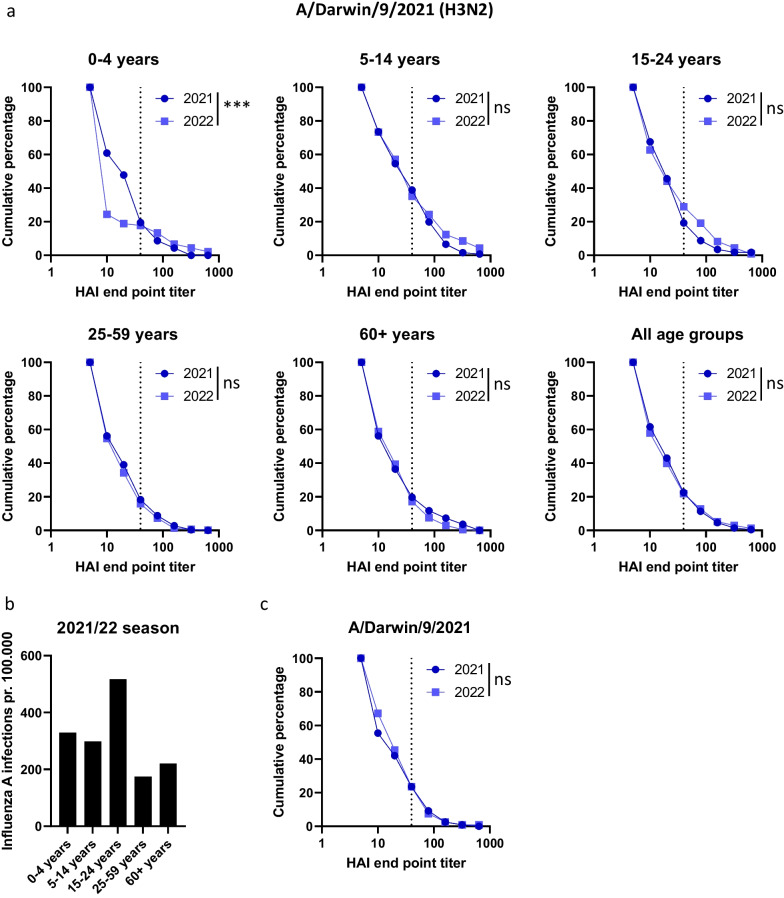
HAI titers against A/Darwin/9/2021(H3N2) in sera from 2021 and 2022 in different age groups. **A** Reverse cumulative plots were generated from the HAI titers against A/Darwin/9/2021 from 2021 and 2022 for the age groups 0–4 years, 5–14 years, 15–24 years, 25–59 years and 60 + years. The dotted line indicates 50% protective HAI titer of 40. **B** Number of detected influenza A infections per 100.000 individuals in the different age groups were extracted from the Norwegian Laboratory Database for the period week 1 2022 to week 26 2022. **C** Reverse cumulative plots of HAI titers in 119 sera collected in 2021 from one reference lab tested against A/Darwin/9/2021 in both 2021 and 2022 to verify reproducibility. Significant differences were calculated by a two-tailed Mann–Whitney test. ***p < 0.001 and ns = no significant difference

To better evaluate the changes in HAI titers from one season to the next we generated reverse cumulative plots for different age groups for sera collected in 2021 and 2022 seasons which were tested against the same A(H1N1)pdm09 and A(H3N2) strains (Figs. 3A and 4A). Reverse cumulative plots display all the HAI data and makes it possible to identify changes in antibody levels that are not apparent when just evaluating the percentage of sera with titer titers ≥ 40. For the 0–4 years age group, HAI titers were significantly lower against A/Victoria/2570/2019 in 2022 compared to 2021 (Fig. 3A). The data indicate that most children aged 4 or younger did not have any immunity against A(H1N1)pdm09 in August 2022, constituting an immunity gap which may have contributed to the high number of confirmed influenza A infections during the 2022/23 season (Fig. 3B). Similarly, we observed a significant reduction in HAI titers against A/Darwin/9/2021 (H3N2) in the youngest age group, despite the percentage of sera with protective titers being quite stable from 2021 to 2022 (Figs. 2B and 4A).

HAI titers against A/Victoria/2570/2019(H1N1)pdm09 were stable in the 5–14 years and 15–24 years age groups, suggesting limited antibody waning from one season to the next (Fig. 3A). There was a slight increase in HAI titers against A/Darwin/9/2021(H3N2) in the 15–24 years age group in sera from 2022, which may reflect the higher number of infections reported in this age group during spring 2022 (Fig. 4A-B).

In the older age groups, there was a significant increase in HAI titer against A/Victoria/2570/2019(H1N1)pdm09 from 2021 to 2022 likely reflecting the inclusion of this strain in the seasonal influenza vaccine administered in fall 2021. For the 2021/2022 season, vaccine coverage in the + 60 years age group were estimated to be at least 55%, while the coverage in the 25–59 years group were at least 17.4% (Fig. 3C). To ensure that HAI data were comparable when collected and analyzed over two consecutive seasons, a set of 119 sera from one microbiological laboratory collected in 2021 were also analyzed together with the samples collected in 2022. No significant difference was seen in HAI assays performed in 2021 and 2022 for the reference samples against A/Victoria/2570/2019 or A/Darwin/9/2021 (Figs. 3D and 4C).

### Lower HAI titers against A(H1N1)pdm09 clade 6B.1A.5a.2a.1 in 2022

**Fig. 5:**
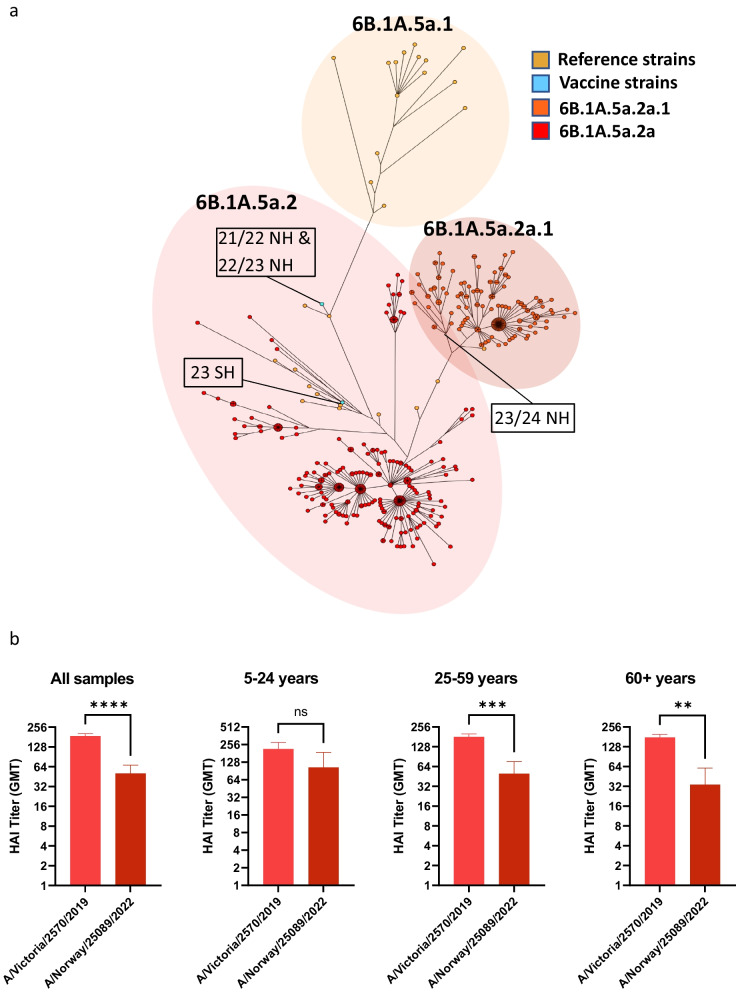
Reduced HAI titers against A(H1N1)pdm09 clade 6B.1A.5a.2a.1 in 2022. **A** Maximum parsimony tree of HA sequences of Norwegian A(H1N1)pdm09 strains from the 2022/2023 influenza season, including reference strains and vaccine strains for the southern and northern hemisphere. **B** Residual serum samples from August 2022 with HAI titer of ≥ 160 against A/Victoria/2570/2019 (clade 6B.1A.5a.2) were evaluated in an HAI assay against the A(H1N1)pdm09 clade 6B.1A.5a.2a.1 strain A/Norway/25089/2022. **B** Data presented is geometric mean with error bars representing 95% confidence interval. Significance was determined using a Wilcoxon matched-paired signed rank test. **p < 0.01, ***p < 0.001 and ****p < 0.0001

The 2022/2023 influenza season predominantly consisted of infections with A(H1N1)pdm09 viruses, although there was increasing numbers of influenza B infection during late winter/spring of 2023. There was cocirculation of A(H1N1)pdm09 viruses belonging to the 6B.1A.5a.2a clade (which include A/Victoria/2570/2019), and the 6B.1A.5a.2a.1 clade including A/Norway/25089/2022 (Fig. 5A). To assess immunity towards the novel A/Norway/25089/2022 strain, we selected 75 serum samples collected in 2022 with HAI titer ≥ 160 against A/Victoria/2570/2019 and tested them in HAI assays against A/Norway/25089/2022 (Fig. 5B). We observed significantly lower HAI titers against the 6B.1A.5a.2a.1 clade with geometric mean titer declining from 187 against A/Victoria/2570/2019 to 50 against A/Norway/25089/2022 (Fig. 5B).

When dividing serum samples into age groups, the reduction in HAI titers against A/Norway/25089/2022 were more prominent in the 25–59 years and 60 + years age groups (Fig. 5B). The reduction in HAI titer in the younger age group did not reach significance. Due to a limited number of serum samples, the 5–14 and 15–24 age groups were combined. None of the serum samples in the 0–4 years age group had HAI titers ≥ 160 against A/Victoria/2570/2019 and thus were not included in the comparison. The marked reduction in HAI titers against A/Norway/25089/2022 in the oldest age group supports the decision of WHO to update the influenza vaccine strain recommendation for 2023/2024 for the northern hemisphere to include a virus of the 6B.1A.5a.2a.1 clade [36].

## Discussion

The long absence of influenza virus during the COVID-19 pandemic have raised concerns about waning immunity and more severe epidemics following the removal of non-pharmaceutical interventions. Indeed, the term “immunity debt” has been used to describe a situation where the lowered immunity in the general population would need to be “repaid” through either infections or vaccinations [37]. However, our results indicate that the immunity against influenza has remained relatively stable in most age groups over the pandemic, and that the two influenza A epidemics of spring 2022 and winter 2022/2023 have been comparable in magnitude to previous seasonal epidemics.

For children in the 0–4 years age group, we observed a pronounced drop in the percentage of sera with protective levels of antibodies against A(H1N1)pdm09 from 2019 until 2022, suggesting that the absence of influenza during the COVID-19 pandemic has generated an immunity gap in this age group which, more than the other groups, is characterized by replacement with many immunologically naïve individuals every year. It should be noted that sera collected in 2019 may have had above-average levels of protective antibodies due to being collected following the extensive A(H1N1)pdm09 epidemic of 2018/2019. In contrast, sera collected in 2022 were predominantly from children who were born after the 2018/2019 influenza season and therefore were antigenically naïve to A(H1N1)pdm09 viruses. Corresponding to the lack of pre-season immunity, we observed a high incidence of influenza A infections in the 0–4 years group during the A(H1N1)pdm09-dominated 2022/2023 winter season. It is possible that testing regimes may have differed between age groups, resulting in a higher number of tests and more confirmed infections in the youngest children. This is, however, contradicted by the fact that a similar elevated incidence in the youngest was not seen for influenza A infections during the A(H3N2) epidemic in spring 2022. The percentage of sera with protective titers against A/Victoria/2570/2019 rose almost fourfold in 2023 following the A(H1N1)pdm09-dominated 2022/2023 season, suggesting that the immunity gap was at least partially closed. Protective levels of antibodies against A(H3N2) were more similar in sera collected in 2019 and 2022, potentially influenced by the late A(H3N2) epidemic in spring of 2022. Nevertheless, disease burden in the youngest children should be carefully monitored over the coming influenza seasons, as they may still be at increased risk of infection and disease from influenza A and B viruses.

For the age groups above 4 years, that have probably had previous exposure to A(H1N1)pdm09 viruses, we observed a more moderate change in protective antibodies from 2019 to 2022. When evaluating sera collected in 2021 and 2022 against the A/Victoria/2570/2019(H1N1)pdm09 strain, HAI titers remained surprisingly stable from one season to the next. Our observations are in accordance with studies in health care workers receiving an AS03-adjuvanted H1N1pdm09 vaccine in 2009 where elevated antibody levels remained for 60 months without additional booster vaccinations or known influenza infection [38]. While the duration of the protective antibody responses following the H1N1pdm09 vaccine may be influenced by the AS03 adjuvant [39], the results indicate that HAI titers after immunization can remain stable when tested against the same strain over time [38]. Similar results were also observed for HAI titers against A/Darwin/9/2021 (H3N2) which remained stable in sera from all age groups from 2021 to 2022. Conceivably, since the sera are collected in late summer, an initial drop in individual post-exposure peak titres down to a more persistent steady-state titre may have already occurred.

In the oldest age groups, we observed an increase in HAI titer against A/Victoria/2570/2019 (H1N1pdm09) from 2021 to 2022, probably reflecting the inclusion of this strain in the seasonal influenza vaccine for the 2021/2022 season which saw very little actual A(H1N1)pdm09 circulation. In Norway, annual influenza vaccination is recommended for people in risk groups, including all above 65 years, which may explain why vaccination rates and the increase in HAI titers are highest in the 60 + age group. However, HAI titers also increased in the 25–59 years group even lthough vaccination coverage was only 17.4% in the general population. Since the residual sera is predominantly collected from microbiological laboratories associated with hospitals, there is a chance that we have a higher frequency of sera from people in various risk groups and consequently higher vaccination coverage than the general population. Previous studies have indicated that HAI titers in the elderly largely return to baseline within 12 months after vaccination [16]. In contrast, our observations indicate that HAI titers may remain significantly elevated after 12 months as the residual sera were collected during the same time period each year. Since the samples are irreversibly anonymized upon collection, we cannot exclude that there are inherent differences between sera collected in 2021 and 2022. However, HAI titers remained the stabile against A/Darwin/9/2021 (H3N2) from 2021 to 2022, and then increased from 2022 to 2023 when the strain was included in the seasonal influenza vaccine for 2022/2023, supporting the idea that vaccine uptake may have improved HAI titers in the older age group.

The stable HAI titers against A/Victoria/2570/2019 and A/Darwin/9/2021 from 2021 to 2022 indicate limited waning between seasons. Consequently, it is highly likely that the reduction in percentage of sera with HAI titers ≥ 40 against contemporaneous strains from 2019 to 2021 is related to antigenic drift due to immune evasive mutations in the HA. Consistent with this conclusion, previous studies have observed reduced effectiveness of the influenza vaccine for the 2019/2020 season (containing A/Brisbane/2/2018, clade 6B.1A.1) against A(H1N1)pdm09 viruses of the 6B.1A.5A clade with additional mutations K130N, N156K, L161I and V250A (as found in A/Victoria/2570/2019) [40]. Similarly, serum samples collected in 2022 with high HAI titers against A/Victoria/2570/2019 displayed a significant reduction in titers against A/Norway/25089/2022, indicating further antigenic drifting of the 6B.1A.5a.2a.1 clade which circulated during the 2022/2023 influenza season. The difference in titers was least for the 5–24-year-olds. This may reflect that the antibody repertoire in younger people is more directed against recent strains than in older people, while less “monospecific” than in the very young who generally have experienced only one strain of the subtype, if any.

Our study of protective immune responses against subtype A(H1N1)pdm09 and A(H3N2) viruses was performed using HAI assays which measures antibodies that block binding of influenza HA to sialic acids on red blood cells. While the assay is recognized as a correlate of protection against influenza infection, it does not measure all protective antibodies that may be induced following infection or vaccination. Antibodies directed against the stem or other epitopes on HA apart from the sialic binding site may neutralize the virus or target infected cells for cellular immune responses and thus contribute to protection [22], but would likely not be measured in our assays. This is also the case for antibodies against neuraminidase (NA) which may protect against infection [41], and has been established as a correlate of protection against influenza [42, 43]. Consequently, the HAI results are likely an underestimate of the protective responses in the population, especially in the age groups that have been exposed to influenza infections over several seasons.

## Conclusion

Analysis of serum samples collected in 2019, 2021, 2022 and 2023 indicate that antibody levels against seasonal influenza A viruses in the Norwegian population have remained relatively stable over the course of the COVID-19 pandemic. The observed reduction in protective antibodies against A(H1N1)pdm09 and A(H3N2) viruses are likely associated with viral antigenic drift. However, due to the absence of influenza viruses for almost two years, an immunity gap developed in the youngest children who were mostly naïve to influenza virus infection by summer 2022. Even though many of these children must be expected to have experienced their first influenza virus infection during the 2022/2023 influenza season, this age group may still have a higher susceptibility to influenza infection in the coming years. Increased focus on vaccination of children with elevated risk of severe infection should therefore be prioritized.

### Supplementary Information

**Additional file 1**. **Figure S1:** Percentage distribution of influenza A and B subtypes from seasons 2008/09 through 2022/23, and **Figure S2:** Phylogeny of A(H1N1)pdm09 and A(H3N2) strain tested by HAI.

## Data Availability

Anonymized data and viral samples can be shared in accordance with the data sharing policy of NIPH.

## Abbreviations

HAI: Hemagglutination inhibition
HA: Hemagglutinin
MSIS: Norwegian surveillance system for communicable diseases
NA: Neuraminidase
NIC Norway: National influenza centre
NIPH: Norwegian institute of public health
RBC: Red blood cells
REK: Regional ethics committee
