# Correction: antigenic drift and immunity gap explain reduction in protective responses against influenza A(H1N1)pdm09 and A(H3N2) viruses during the COVID-19 pandemic: a cross-sectional study of human sera collected in 2019, 2021, 2022, and 2023

**DOI:** 10.1186/s12985-024-02341-x

**Published:** 2024-03-18

**Authors:** Even Fossum, Andreas Rohringer, Torstein Aune, Kjersti Margrethe Rydland, Karoline Bragstad, Olav Hungnes

**Affiliations:** 1 Division of Infection Control, Department of Virology, Norwegian Institute of Public Health, PO Box 222 Skøyen, Oslo, 0213 Norway; 2 Division of Infection Control, Department of Vaccines, Norwegian Institute of Public Health, PO Box 222 Skøyen, Oslo, 0213 Norway

Correction: Fossum et al. Virology Journal (2024) 21:57 10.1186/s12985-024-02326-w.

Following the publication of the original article [1], the author reported that the figures had been mistakenly reordered during publication:

Fig. 1 was presented as Fig. 4

Fig. 2 was presented as Fig. 5

Fig. 3 was presented as Fig. 1

Fig. 4 was presented as Fig. 2; and

Fig. 5 was presented as Fig. 3.

The correct order is as follows:

**Fig. 1:**
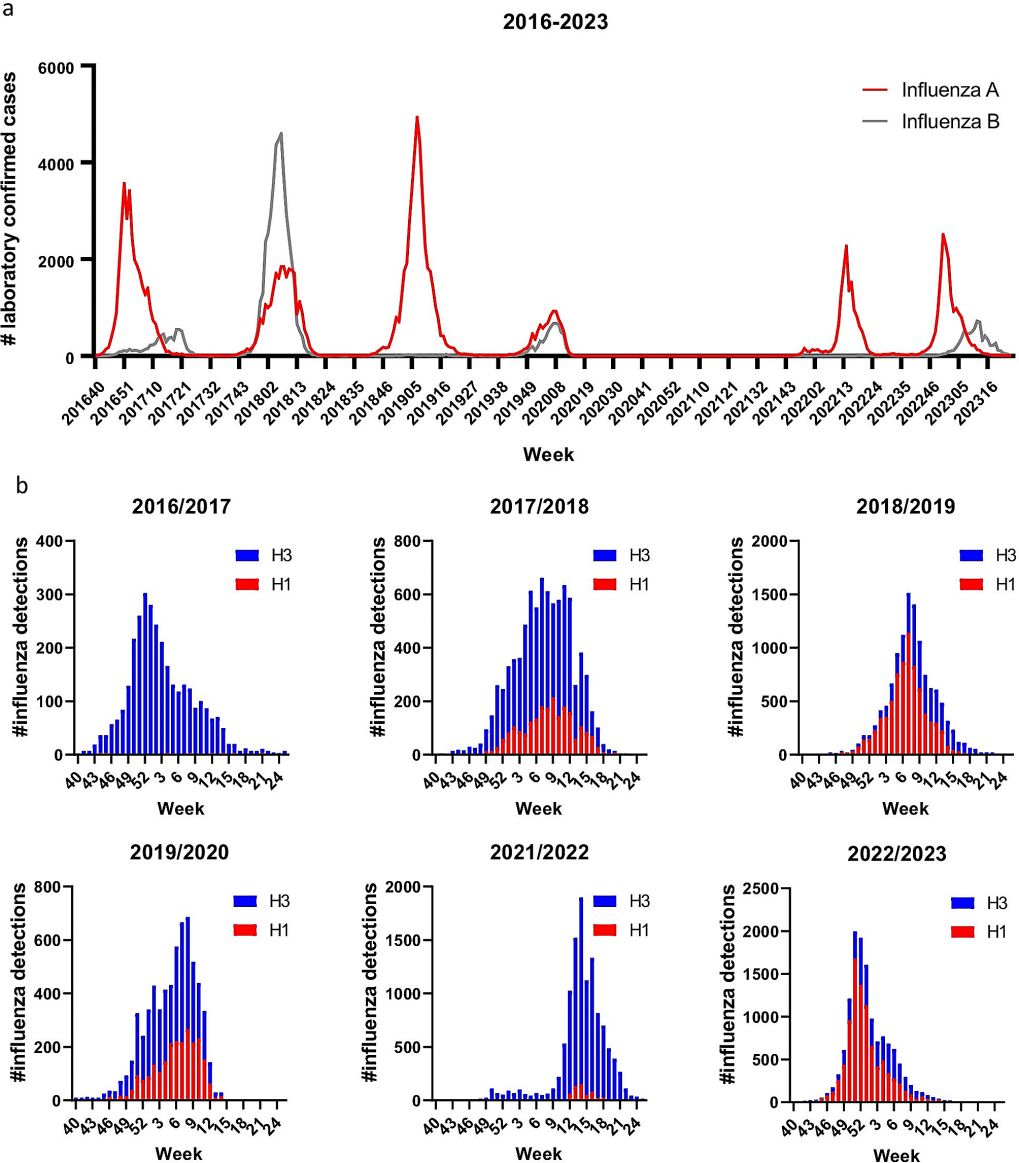
Influenza epidemics in Norway from October 2016 to May 2023. **A**) The number of laboratory confirmed influenza A and B infections were extracted from the Norwegian influenza surveillance data generated by NIPH and the regional laboratories. **B**) The proportions of A(H1N1)pdm09 and A(H3N2) among influenza A infections were estimated from patient samples tested against both subtypes, and these weekly frequencies extrapolated to the total number of detected influenza A infections. Each influenza season is indicated with a start in week 40 and end in week 25 the following year.

**Fig. 2:**
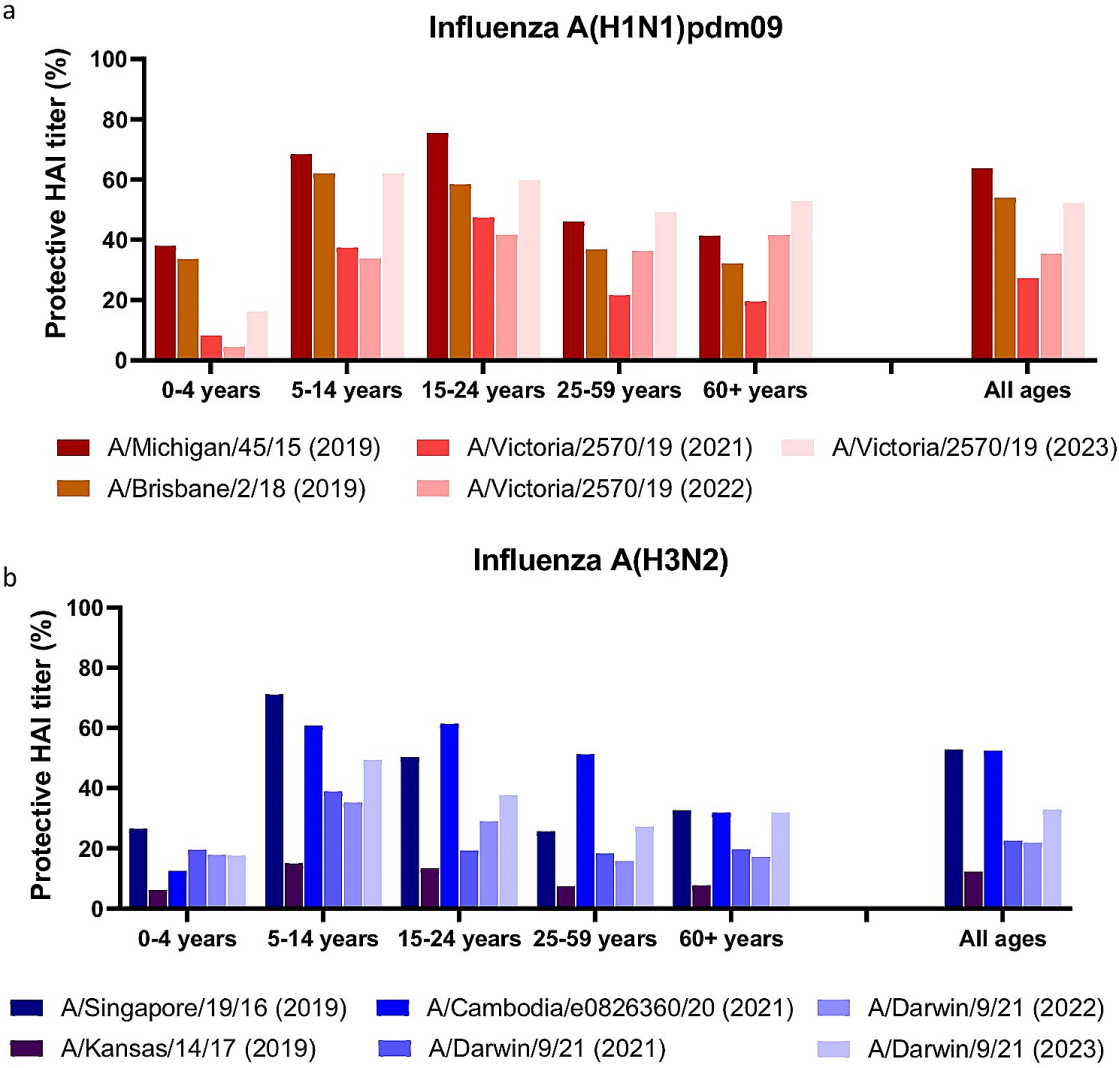
Protective antibodies against influenza A in sera from 2019, 2021, 2022 and 2023. Residual sera collected in August 2019 (n = 1054), 2021 (n = 657), 2022 (n = 1197) and 2023 (n = 456) were evaluated in HAI assay against **A**) H1N1pdm09 strains A/Michigan/45/2015, A/Brisbane/2/2018 or A/Victoria/2570/2019 or **B**) H3N2 strains A/Singapore/19/2016, A/Kansas/14/2017, A/Cambodia/e0826360/2020 or A/Darwin/9/2021. Sera were considered protective if HAI titers were ≥ 40, and the percentage of protective-titre sera plotted in different age groups

**Fig. 3:**
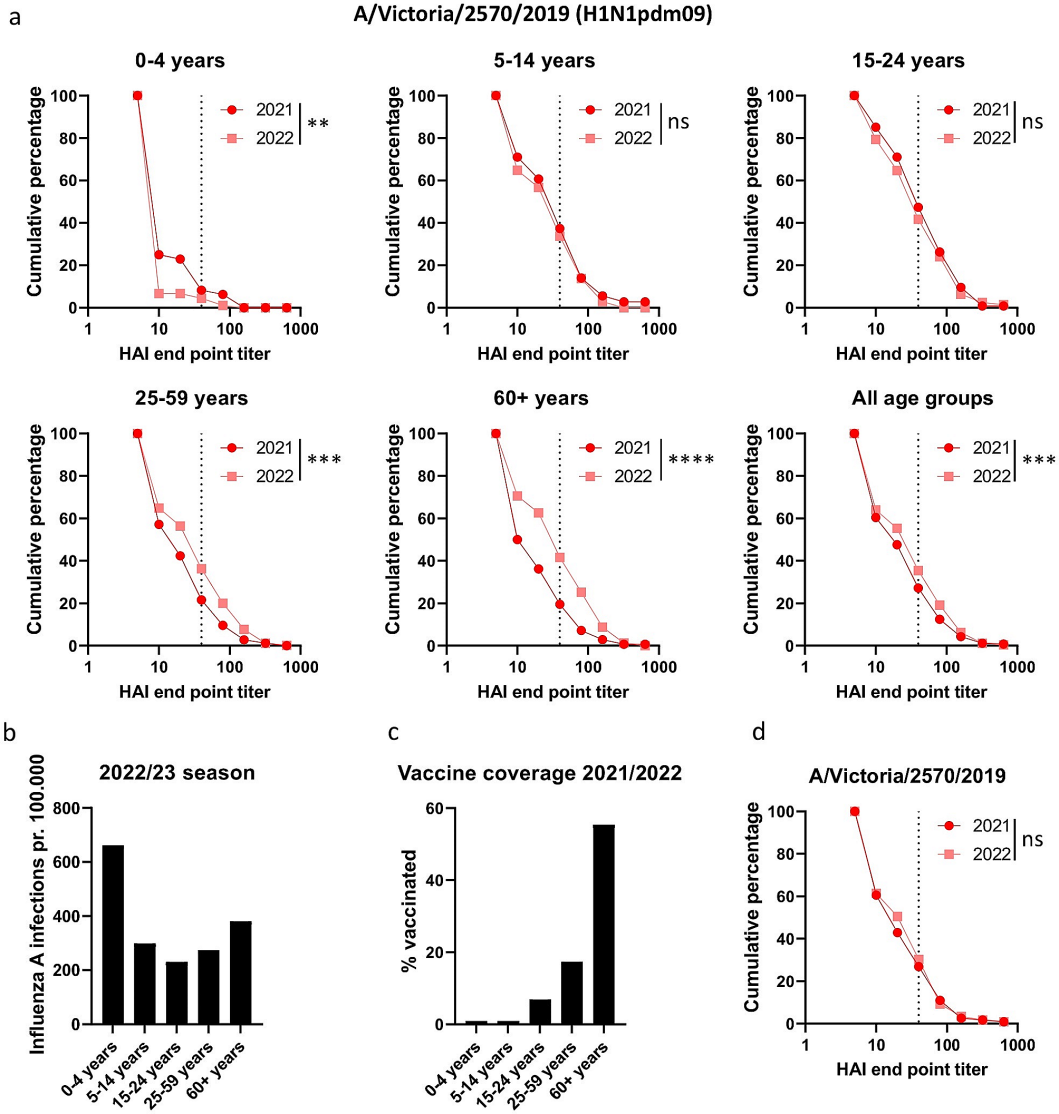
HAI titers against H1N1pdm09 A/Victoria/2570/2019 in sera from 2021 and 2022 in different age groups. **A**) Reverse cumulative plots were generated from the HAI titers against A/Victoria/2570/19 from 2021 and 2022 for the age groups 0–4 years, 5–14 years, 15–24 years, 25–59 years and 60 + years. The dotted line indicates 50% protective HAI titer of 40. **B**) Number of detected influenza A infections per 100.000 individuals in the different age groups for the period week 40 2022 to week 22 2023. **C**) Vaccine coverage in the general population obtained from the Norwegian Immunization Registry SYSVAK for the different age groups. **D**) Reverse cumulative plots of HAI titers in a panel of 119 sera collected in 2021 and first tested against A/Victoria/2570/2019 in 2021 and repeat tested in 2022 to verify reproducibility. A) Significant differences were calculated by a two-tailed Mann-Whitney test. ** = p < 0.01, *** = p < 0.001 and**** = p < 0.0001, ns = no significant difference

**Fig. 4:**
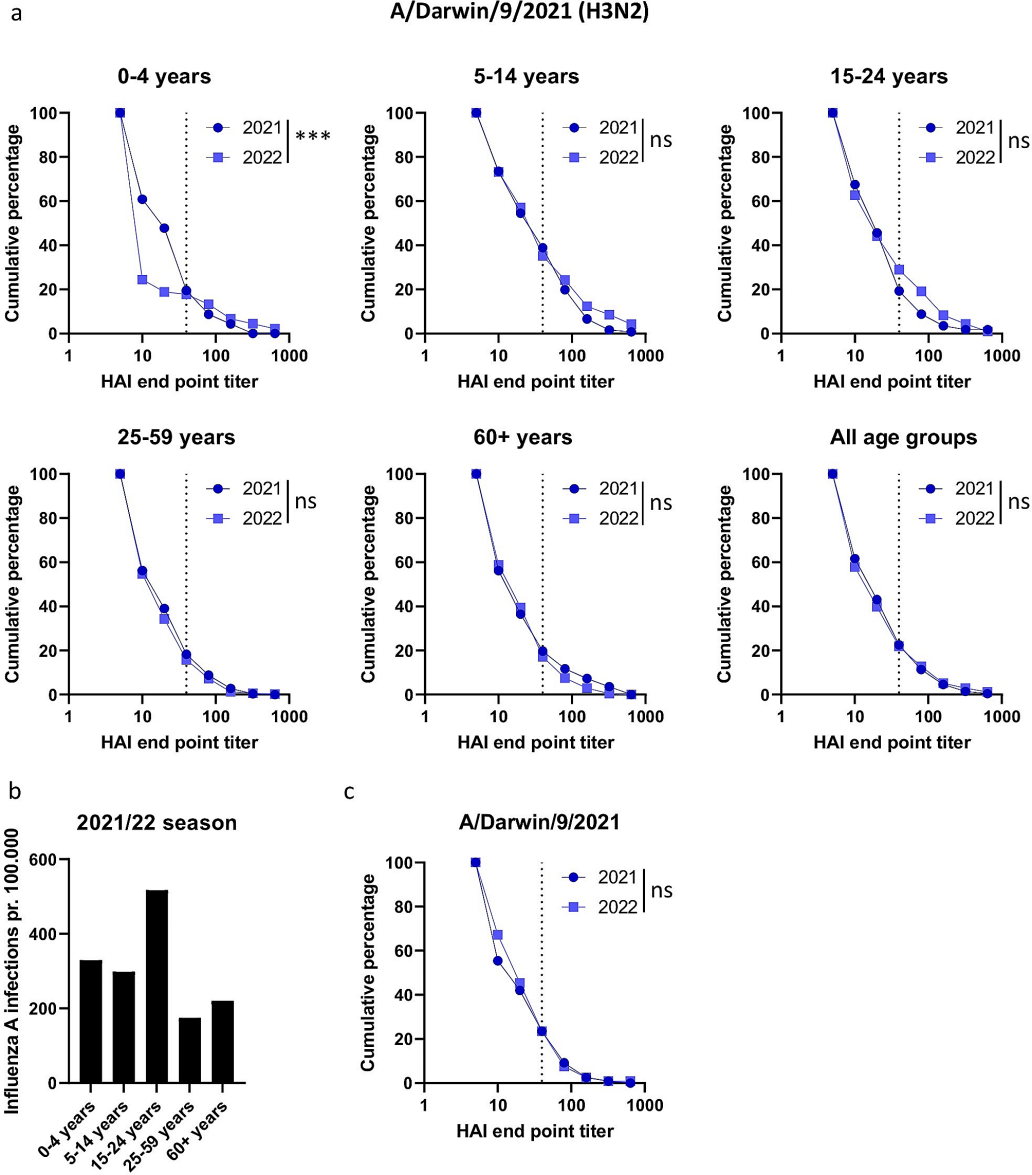
HAI titers against A/Darwin/9/2021(H3N2) in sera from 2021 and 2022 in different age groups. **A**) Reverse cumulative plots were generated from the HAI titers against A/Darwin/9/2021 from 2021 and 2022 for the age groups 0–4 years, 5–14 years, 15–24 years, 25–59 years and 60 + years. The dotted line indicates 50% protective HAI titer of 40. **B**) Number of detected influenza A infections per 100.000 individuals in the different age groups were extracted from the Norwegian Laboratory Database for the period week 1 2022 to week 26 2022. **C**) Reverse cumulative plots of HAI titers in sera from one reference lab tested against A/Darwin/9/2021 in both 2021 and 2022. Significant differences were calculated by a two-tailed Mann-Whitney test. *** = p < 0.001 and ns = no significant difference

**Fig. 5:**
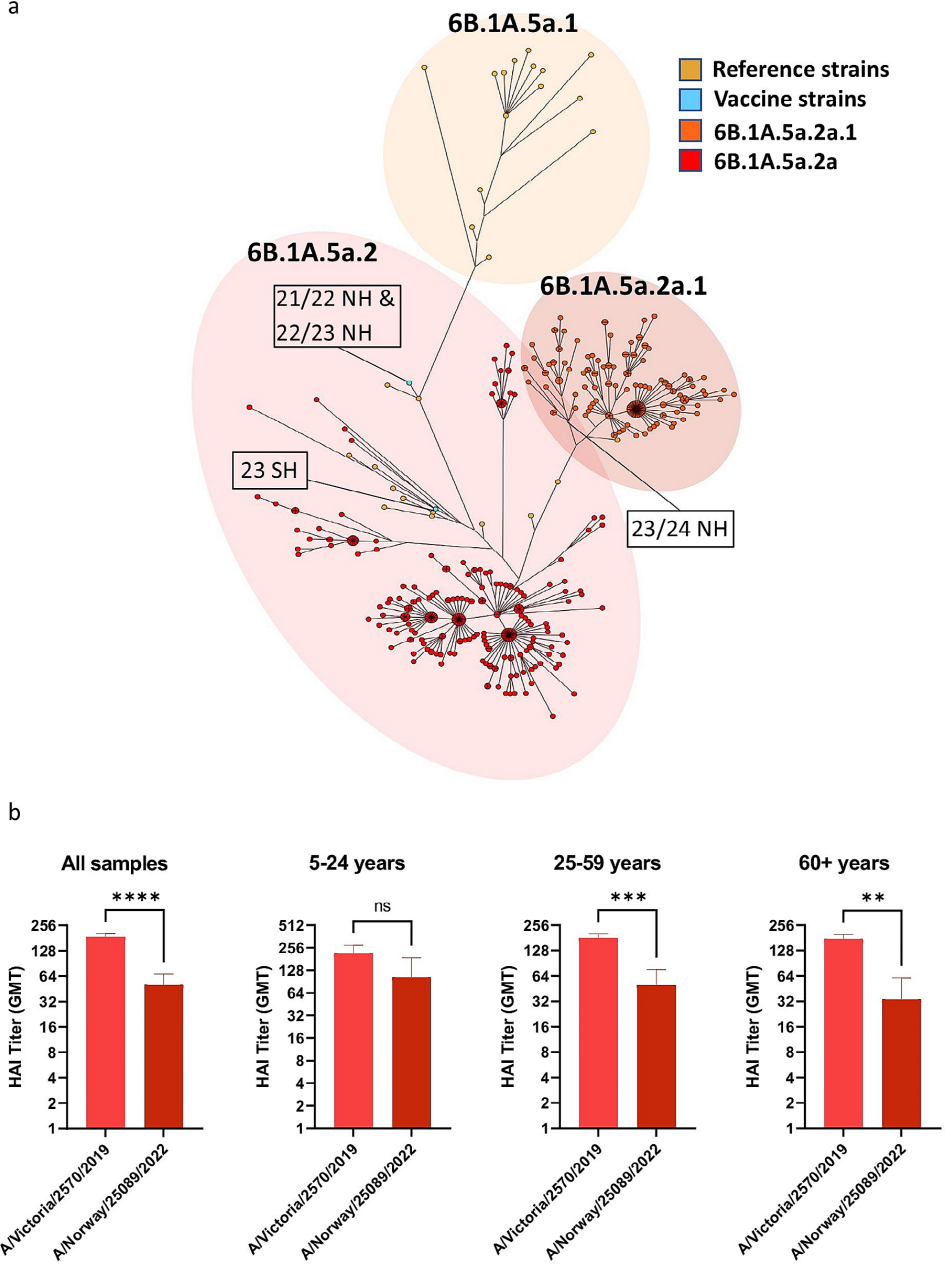
Reduced HAI titers against A(H1N1)pdm09 clade 6B.1A.5a.2a.1 in 2022. **A**) Maximum parsimony tree of HA sequences of Norwegian A(H1N1)pdm09 strains from the 2022/2023 influenza season, including reference strains and vaccine strains for the southern and northern hemisphere. **B**) Residual serum samples from August 2022 with HAI titer of ≥ 160 against A/Victoria/2570/2019 (clade 6B.1A.5a.2) were evaluated in an HAI assay against the A(H1N1)pdm09 clade 6B.1A.5a.2a.1 strain A/Norway/25089/2022. **B**) Data presented is geometric mean with error bars representing 95% confidence interval. Significance was determined using a Wilcoxon matched-paired signed rank test. ** = p < 0.01, *** = p < 0.001 and**** = p < 0.0001

The legends shown in the Original Article are correct for all the figures.

The original article [1] has been corrected.
