# Supplementary material for: Antigenic drift and immunity gap explain reduction in protective responses against influenza A(H1N1)pdm09 and A(H3N2) viruses during the COVID-19 pandemic: a cross-sectional study of human sera collected in 2019, 2021, 2022, and 2023
Source: Virol J. 2024 Mar 6;21:57. doi: 10.1186/s12985-024-02326-w (PMC10916265; doi:10.1186/s12985-024-02326-w)

Supplementary Figure 1: Percentage distribution of influenza A and B subtypes from seasons 2008/09 through 2022/23. The distribution of influenza A and B subtypes were generated based on subtype/lineage specific testing performed at the NIPH.

Supplementary Figure 2: Phylogeny of A(H1N1)pdm09 and A(H3N2) strain tested by HAI. Phylogenetic trees based on the hemagglutinin-coding sequences of A) A(H1N1)pdm09 and B) A(H3N2) viral strains analyzed by HAI Figure 2, in addition to selected strains used in seasonal influenza vaccines. The trees were constructed in MEGA11 using the Neighbour-Joining method. The H1N1 tree was rooted in A/Christchurch/16/2010, while the H3N2 tree was rooted in A/Texas/50/2012. The clade associated with each influenza strain is indicated to the left of the trees.

Supplementary Figure 1:

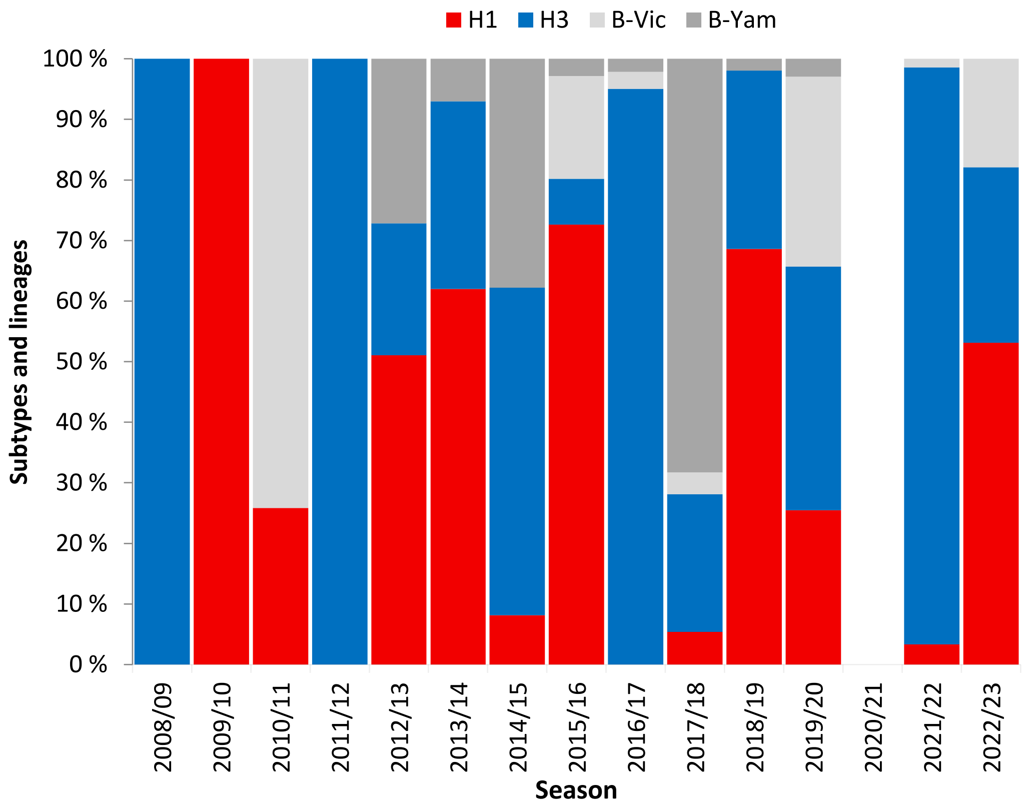

Supplementary Figure 2:

A

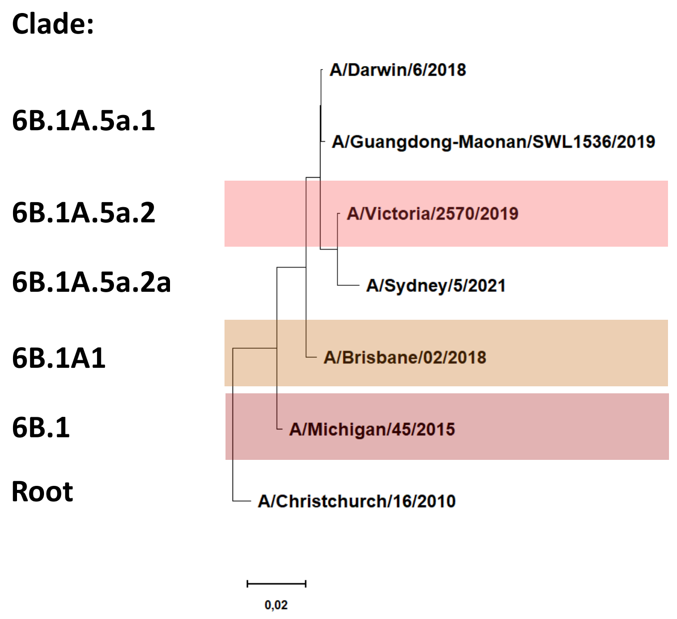

B

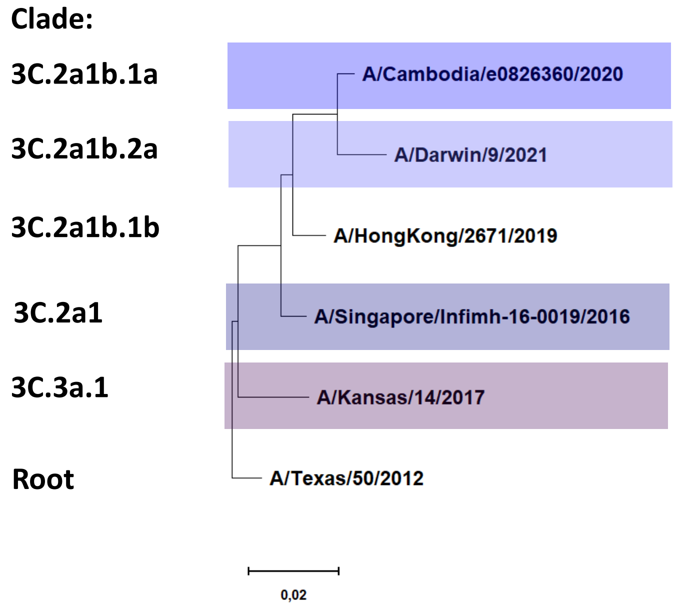

Supplement: Supplementary file 1 — Additional file 1. Figure S1: Percentage distribution of influenza A and B subtypes from seasons 2008/09 through 2022/23, and Figure S2: Phylogeny of A(H1N1)pdm09 and A(H3N2) strain tested by HAI. [file 12985_2024_2326_MOESM1_ESM.pdf]
